# Protective Effect of the LRRK2 Kinase Inhibition in Human Fibroblasts Bearing the Genetic Variant GBA1 K198E: Implications for Parkinson’s Disease

**DOI:** 10.1007/s12017-025-08864-y

**Published:** 2025-05-21

**Authors:** Laura Patricia Perez-Abshana, Miguel Mendivil-Perez, Carlos Velez-Pardo, Marlene Jimenez-Del-Rio

**Affiliations:** 1 Neuroscience Research Group, Faculty of Medicine, Institute of Medical Research, University of Antioquia, University Research Headquarters, Calle 70 #52-21 and Calle 62#52-59, Building 1, Laboratory 411/412, Medellin, 050010 Colombia; 2 Faculty of Nursing, University of Antioquia, University Research Headquarters, Calle 70 #52-21 and Calle 62#52-59, Building 1, Laboratory 411/412, Medellin, 050010 Colombia

**Keywords:** LRRK2, RAB10, Fibroblasts, GBA, Parkinson’s disease, PF-06447475

## Abstract

Parkinson's disease (PD) is a chronic and progressive neurodegenerative disorder for which there are currently no curative therapies. Therefore, the need for innovative treatments for this illness is critical. The glucosylceramidase beta 1 (GBA1) and leucine-rich repeated kinase 2 (LRRK2) genes have been postulated as potential genetically defined drug targets. We report for the first time that the LRRK2 inhibitor PF-06447475 (PF-475) not only restores GCase enzyme activity, but also increases mitochondrial membrane potential, significantly decreases DJ-1 Cys106-SO_3_, reduces lysosome accumulation, and diminishes cleaved caspase-3 (CC3) in GBA1 K198E fibroblasts. Furthermore, in addition to a significant reduction in p-Ser935 LRRK2 kinase, we found that PF-475 reduced p-Thr73 RAB 10 and p-Ser129 α-Syn in mutant skin fibroblasts. In addition, we found that the GCase activator GCA (NCGC00188758) increased GCase activity and decreased lysosomal accumulation, but did not affect p-Ser935 LRRK2, ∆Ψm, p-Ser129 α-Syn, DJ-1 Cys106-SO_3_, or CC3 in K198E GBA1 fibroblasts. The GCase inhibitor conduritol-β-epoxide (CBE), used as an internal control, significantly reduced GCase and left the other pathological markers largely unaltered in GBA1 K198E, but reduced GCase and increased the accumulation of lysosomes only in WT GBA1 fibroblasts. Taken together, these results suggest that LRRK2 is a critical signaling kinase in the pathogenic mechanism associated with the lysosomal GBA1/GCase K198E variant. Our findings suggest that the use of LRRK2 inhibitors in PD patients with GBA1 mutations, such as K198E, may be effective in reversing GBA1/GCase deficiency, autophagy impairment, oxidative stress, and neuronal death.

**Graphical Abstract:**

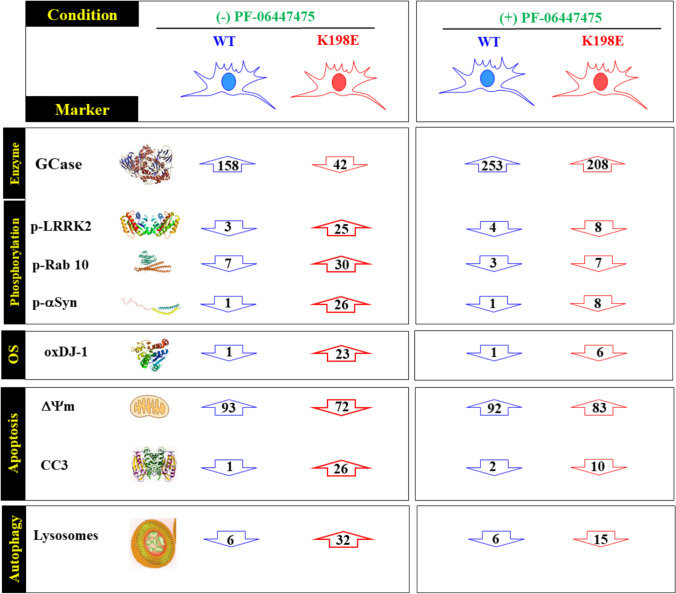

## Introduction

Parkinson's disease (PD) is a chronic and progressive neurodegenerative disorder mainly characterized by resting tremor, bradykinesia, rigidity, and loss of postural reflexes (Váradi, 2020) caused by the deterioration of dopaminergic (DAergic) neurons from the pars compacta of the substantia nigra (Forno, 1996). Pathologically, affected neurons are recognized by the presence of Lewy's intraneuronal inclusion bodies (Engelhardt and Gomes 2017; Goedert et al., 2013), which consist of protein aggregates of alpha-synuclein (α-Syn) (Spillantini et al., 1998). Several molecular genetic reports have demonstrated that genetic factors play an important role in the development of sporadic and familial PD. Indeed, studies of familial PD have identified at least 20 different causative genes, and genome-wide association studies have identified more than 200 genes as potential drivers of PD development (Day & Mullin, 2021; Funayama et al., 2023). Despite this overwhelming picture, glucosylceramidase beta 1 (GBA1) and leucine-rich repeated kinase 2 (LRRK2) genes have been postulated to be involved in all forms of PD and may play a broader role in PD pathogenesis (Pang et al., 2022). Furthermore, there is crosstalk between LRRK2 and GBA in PD (Lee et al., 2021); however, the exact mechanism is unknown. Therefore, it is imperative to investigate the molecular mechanism of action of GBA1 (Chatterjee & Krainc, 2023; Pradas & Martinez-Vicente, 2023; Zhang et al., 2024) and LRRK2 (Berwick et al., 2019; Rocha et al., 2022; Sosero & Gan-Or, 2023). This premise takes on even greater significance as genetically defined drug targets show great promise (Maayan Eshed & Alcalay, 2024; Zhang et al., 2024) and clinical trials targeting the GBA1 and LRRK2 genes are underway (e.g., Heijer et al., 2023; Kingwell, 2023).

The glucosidase beta acid 1 (GBA1) gene encodes the β-glucocerebrosidase (GCase) enzyme (Horowitz et al., 1989). GCase is a functional lysosomal hydrolase with 497 amino acids (a.a.) that cleaves glucosylsphingosine (GlcSph) to glucose and sphingosine or glucosylceramide (GlcCer) to glucose and ceramide (KEGG Enzyme EC 3.2.1.45). Structurally, GCase consists of four discrete domains. Domain I (residues 1–27 and 383–414) is a three-stranded antiparallel β-sheet surrounded by a loop and a perpendicular strand. Domain II (residues 30–75 and 431–497) has two closely related β-sheets that form an independent domain resembling an immunoglobulin (Ig) fold. Domain III (residues 76–381 and 416–430) is a (β/α)_8_ triosephosphate isomerase (TIM) barrel with three cysteines at residues 126, 248, and 342 that serves as the catalytic site of the enzyme (Dvir et al., 2003; Liou et al., 2006; Smith et al., 2017; Smith et al., 2022). Crucially, within the lysosome, the substrate-presenting cofactor saposin C, a necessary 84-residue activator peptide, is required for GCase activation (Tamargo et al., 2012). According to the Human Gene Mutation Database (http://www.hgmd.org, accessed January 2025), there are currently 716 known disease-causing GBA1 mutations, including 535 missense/nonsense, 42 splicing, and 5 regulatory mutations; 55 and 20 small deletions and small insertions, respectively; 9 indels; 15 and 3 large deletions and large insertions, respectively; and 32 complex mutations. However, the pathogenic mechanisms of PD associated with GBA1 mutations are not fully understood. Interestingly, among the different missense mutations documented so far, we found that PD patients have a significantly higher percentage of GBA1 mutant carriers than healthy controls, mainly due to the presence of a population-specific variant located in Colombia, the GBA1 p.K198E (Velez-Pardo et al., 2019). This nonsense mutation is a single base substitution of a lysine (*K*) for a glutamic acid (*E*) at codon 198 (c.709 A > G, g.4385 A > G, exon 6, p.Lys198Glu) (Velez-Pardo et al., 2019). In fact, heterozygous p.K198E (hereafter K198E) is a significant risk factor for PD (Velez-Pardo et al., 2019), while homozygous K198E seems to be associated with Gaucher disease (GD) type 2 (infantile, neuropathogenic) (Orvisky et al., 2002). Molecularly, the GBA1 K198E variant has been shown to be associated with suppression of GCase activity, autophagy impairment, oxidative stress (OS), mitochondrial damage, and apoptosis in skin fibroblasts (Perez-Abshana et al., 2024). Specifically, K198E was found to be associated with abnormal phosphorylation of the proteins LRRK2 and α-synuclein at pathological residues Ser935 and Ser129, respectively. However, whether the use of GCase activators (e.g., NCGC00188758 or GCA) (Patnaik et al., 2012) could correct the K198E GCase deficiency is not yet known.

LRRK2 is a 288 kDa polypeptide with a number of unique structural domains, including leucine-rich repeats (LRR), ankyrin (ANK) repeats, armadillo (ARM) repeats, a short (19-residue) hinge helix, an atypical Roco family GTPase domain consisting of a catalytic kinase (KIN) domain, a β propeller (WD40) toroid, an extended (29-residue) C-terminal αC helix, and a GTP-binding Ras of complex proteins (ROC) domain coupled to two tandem folds C-terminal to ROC (COR-A and COR-B) (Alessi & Pfeffer, 2024). To date, 988 public variants have been published (https://databases.lovd.nl/shared/genes/LRRK2, accessed January 2025). About 80% of people with LRRK2 mutations are affected by the G2019S mutation, which is the most common pathogenic mutation of LRRK2 in humans (Simpson et al., 2022). It affects the glycine in the Asp-Tyr-Gly (DYG) magnesium-binding motif within the kinase domain. Although the specific molecular mechanism leading to dopaminergic (DAergic) neuronal death in PD is not fully understood (Absalyamova et al., 2025), several studies have shown that LRRK2 is associated with increased susceptibility to OS, mitochondrial depolarization, and cell death (Angeles et al., 2011; Heo et al., 2010; Mendivil-Perez et al., 2016; Nguyen et al., 2011; Quintero-Espinosa et al., 2017; Singh et al., 2019; West et al., 2007). While the LRRK2 kinase domain has been implicated in OS-induced neurotoxicity (Heo et al., 2010; Mendivil-Perez et al., 2016; Quintero-Espinosa et al., 2017), it is not yet clear how the LRRK2 protein is linked to lysosomal GBA1/GCase. Nevertheless, abnormally phosphorylated p-Ser935 LRRK2 appeared concomitantly with GCase enzyme deficiency, oxidized DJ-1, p-Ser129 α-Syn, mitochondrial damage, accumulation of lysosomes and autophagosomes, and cleaved caspase-3 (CC3) (Perez-Abshana et al., 2024). In addition, LRRK2 phosphorylates RBA10, a key protein regulator of cellular vesicle trafficking (Ito et al., 2016; Kania et al., 2023; Mamais et al., 2024). Interestingly, pharmacological or genetic ablation of LRRK2 protects against PD-associated environmental toxicants (Ilieva et al., 2024; Mendivil-Perez et al., 2016; Quintero-Espinosa et al., 2017; Quintero-Espinosa et al., 2024; Quintero-Espinosa et al., 2023) and attenuates α-Syn gene-induced PD (Daher et al., 2015). However, whether pharmacological inhibition of LRRK2 could protect DAergic neurons from GBA1 K198E-induced toxicity remains to be determined. Therefore, research on LRRK2 and GBA1 may be essential to understand the biology underlying neuronal degeneration in Parkinson's disease. We hypothesize that pharmacological inhibition of LRRK2 could reverse the pathogenic phenotype associated with the GBA1 K198E variant (Perez-Abshana et al., 2024). This approach may be informative not only for the K198E variant but also for other GBA1 mutations.

To test this premise, we treated either wild-type or GBA1 K198E skin fibroblasts, which are a valid model of PD (Deus et al., 2020; Teves et al., 2018), with the specific LRRK2 inhibitor PF-06447475 (hereafter PF-475) (Henderson et al., 2015). Following biochemical, flow cytometric, and fluorescence microscopic analysis, we found that PF-475 not only restores GCase enzyme activity, but also restores vital cellular functions such as increasing mitochondrial membrane potential (ΔΨm), significantly reducing DJ-1 Cys106-SO_3_, decreasing lysosome accumulation, and shortening CC3 in K198E fibroblasts. Furthermore, in addition to a significant reduction in p-Ser935 LRRK2 kinase, we found that PF-475 reduced p-Thr73 RAB 10 and p-Ser129 α-Syn. We also report for the first time that the enzymatic GCase activator GCA (NCGC00188758) restores GCase enzyme activity and reduces lysosome accumulation in GBA1 K198E fibroblasts. Taken together, these results suggest that LRRK2 plays a critical signaling kinase role in the pathogenic mechanism associated with the lysosomal GBA1/GCase K198E variant. Our findings support the view that pharmacological treatment of Parkinson's patients with inhibitors of LRRK2 may alleviate the symptoms of this neurological disorder (Zhu et al., 2024).

## Materials and Methods

### Human Dermal Fibroblast Culture

Fibroblasts were obtained from skin biopsies of one PD patient carrying a heterozygous GBA1 mutation (K198E, Tissue Bank Code (TBC) # COP0826, male, age at onset 33 years, age at sampling 58 years old) and one healthy control (TBC # 10,624, male, age at sampling 58 years old) matched for age and gender. This study was approved by the Ethics Committee of Research from “Sede de Investigación Universitaria-SIU” (approval code 19-10-845). To isolate dermal fibroblasts, 4 mm skin biopsies of the donors were obtained using a biopsy punch. Skin tissue was cut up into small pieces (< 1 mm), placed into gelatin-coated 6-well dishes (Costar Corning Incorporated), and left to dry at 37 °C until attachment. Once the explants were attached to the plate, culture medium (Dulbecco’s Modified Eagle’sInt. J. Mol. Sci. 2024, 25, 9220 19 of 26 Medium; DMEM, cat #D0819, Sigma, Saint Louis, MO, USA) supplemented with 10% fetal bovine serum (FBS, cat #CVFSVF00-01, Eurobio Scientific, Paris, France) and 1% penicillin/streptomycin (P/S; Gibco) (10% DMEM) was carefully added, and the plates were incubated at 37 °C in a humidified atmosphere with 5% CO_2_. When cells sprouted from the explants, after one week in culture, the plates were washed with PBS to eliminate non-adherent cells and surplus explants, and new 10% DMEM culture medium was added. The culture medium was then replaced every 3–4 days. When sprouted cells reached 80% confluency, subculturing was performed for cell expansion.

### Analysis of Cells

WT and GBA1 K198E fibroblasts were cultured in DMEM with high glucose (4500 mg/L)

plus 10% fetal bovine serum (FBS).

#### Treatment Conditions

Wild-type (WT) and GBA1 K198E fibroblasts at passage 4–6 were treated with or without PF-06447475 (PF-475, 1 µM), an LRRK2 inhibitor (Sigma, Cat# PZ0248), for 24 h. For the present experimental approach, we chose 1 μM PF-475 on the basis of the previous observation that this concentration of inhibitor was effective in blocking of LRRK2 in neuron-like cells (Mendivil-Perez et al., 2016). We use as a control the GCase stabilizer NCGC00188758 (CID de pubchem: 46,907,762), at 10 µM for 24 h (GCA, Sigma-aldrich, 5.31660, Calbiochem, St. Louis MO, United States). GCA -a pyrazolo [1,5-a]pyrimidine [n-(4-ethynylphenyl)−5,7-dimethylpyrazolo[1,5-a] pyrimidine-3-carboxamide] has demonstrated biochemical activation of glucocerebrosidase and chaperone activity in fibroblasts from Gaucher disease (GD) patients. Additionally, cells were treated with 10 µM of the GCase inhibitor conduritol-β-epoxide (CBE, Cayman Chemical company, cat#15,216, medchem express cat#hy-100944) for 24 h. CBE was reconstituted at 10 mg/ml in Dulbecco’s phosphate-buffered saline (DPBS, corning cat#21–031-cv) and stored at − 20 °C. For cell culture experiments, the vehicle or CBE was further diluted in cell media to achieve the desired final concentration.

### Glucocerebrosidase (GCase) Activity Assay

Cellular GCase activity was determined using the Beta-Glucosidase Assay Kit (Abcam, Boston, MA, USA, Cat. ab272521) according to the manufacturer’s recommendations with minor modifications. Briefly, WT and K198E untreated fibroblast cell lysates were incubated for 24 h at 37 °C with p-nitrophenyl-α-D-glucopyranoside, which was hydrolyzed specifically by β-glucosidase into a yellow-colored product (maximal absorbance at 405 nm)

The rate of the reaction was directly proportional to the enzyme activity.

### Cellular Analysis

#### Flow Cytometry and Fluorescent Microscopy Immunofluorescence

After each treatment, cells (1 × 10^5^) were carefully detached and fixed in 80% ethanol and stored at 20 °C overnight. Then, cells were washed with PBS and permeabilized with 0.2% triton X-100 (Cat# 93,443, Sigma-Aldrich, St. Louis, MO, USA) plus 1.5% bovine serum albumin (BSA, Cat# A9418, Sigma-Aldrich, St. Louis, MO, USA) in phosphate-buffered solution (PBS) for 30 min. Then, cells were washed and incubated with primary antibodies (1:200; diluted in PBS containing 0.1% BSA) against p-(S935)-LRRK2 (Abcam cat #AB133450; Boston, MA, USA), α-synuclein (pS129; Abcam cat #AB51253; Boston, MA, USA), oxidized DJ-1 (1:500; ox (Cys106) DJ-1; spanning residue C106 of human PARK7/DJ-1; oxidized to produce cysteine sulfonic (SO3) acid; Abcam cat #AB169520; Boston, MA, USA), cleaved caspase-3, (CC3; 1:250; cat# AB3623, Millipore, Merck, Darmstadt, Germany), and anti-RAB10 (phospho-T73) (Rab10, Abcam, ab241060; 1:100 for ICC and 1:1000 for WB), overnight at 4 °C. After exhaustive rinsing, we incubated the cells with secondary fluorescent antibodies (DyLight 488 horse anti-rabbit and mouse antibodies, cats DI 1094 and DI 2488, Vector Laboratories, Newark, NJ, USA) at 1:500. Finally, cells were washed and re-suspended in PBS for analysis on a BD LSRFortessa II flow cytometer (BD Biosciences, Franklin Lakes, NJ, USA). Twenty-thousand events were acquired, and the acquisition analysis was performed using FlowJo 7.6.2 Data Analysis Software (BD Biosciences, Franklin Lakes, NJ, USA). For fluorescence microscopy analysis, attached cells were fixed in 80% ethanol and incubated with primary and secondary antibodies as described above. Nuclei were stained with Hoechst 33,342 (0.5 µM) and fluorescence microscopy photographs were taken using a Zeiss Axio Vert.A1 Fluorescence Microscope equipped with a Zeiss AxioCam Cm1.

#### Characterization of Lysosomal Complexity

To analyze lysosomal complexity, cells were incubated with the cell-permeable, nonfixable, green, fluorescent dye LysoTracker Green DND-26 (50 nM, cat #L7526, Thermo Fisher Scientific, Waltham, MA, USA) for 30 min at 37 °C. Then, the cells were washed, and LysoTracker fluorescence was determined by flow cytometry using a BD LSRFortessa II flow cytometer (BD Biosciences, Franklin Lakes, NJ, USA) or fluorescent microscopy using a Zeiss Axio Vert.A1 Fluorescence Microscope equipped with a Zeiss AxioCam Cm1. The experiment was conducted three times, and 20,000 events were acquired for analysis. Flow cytometry analysis for LysoTracker was performed by selecting, in the FL-1 channel, all cells with LysoTracker reactivity (> 99%). Quantitative data and figures were obtained using FlowJo 7.6.2 Data Analysis Software (BD Biosciences, Franklin Lakes, NJ, USA).

#### Analysis of Mitochondrial Membrane Potential *(∆Ψm*)

Assessment of the *∆Ψm* was performed according to (Monteiro et al., 2020). We incubated cells for 20 min at RT in the dark with a deep red MitoTracker (20 nM final concentration) compound (Thermo Scientific, cat# M22426, Netherlands, Europe). Cells were analyzed with flow cytometry using a BD LSRFortessa II flow cytometer (BD Biosciences, Franklin Lakes, NJ, USA) or fluorescent microscopy using a Zeiss Axio Vert.A1 Fluorescence Microscope equipped with a Zeiss AxioCam Cm1. The experiment was conducted three times, and 20,000 events were acquired for analysis. Quantitative data and figures were obtained using FlowJo 7.6.2 Data Analysis Software.

#### Data Analysis

In this experimental design, two vials of fibroblast were thawed (WT GBA1 and GBA1 K198E), cultured, and the cell suspension was pipetted at a standardized cellular density of 2 × 10^4^ cells/cm^2^ into different wells of a 24- or 6-well plate. Cells (i.e., the biological and observational units) (Lazic et al., 2018) were randomized to wells by simple randomization (sampling without replacement method), and then wells (i.e., the experimental units) were randomized to treatments by a similar method. Experiments were performed on three independent occasions (*n* = 3) blind to the experimenter and/or flow cytometer analyst (Lazic et al., 2018). The data from the three repetitions, i.e., independent experiments, were averaged, and representative flow cytometry density or histogram plots from the three independent experiments were selected for illustrative purposes, whereas the bars in the quantification figures represent the mean ± S.D. and the three black dots show the data point of each experimental repetition. Based on the assumptions that the experimental unit (i.e., the well) data comply with the independence of observations, the dependent variable is normally distributed in each treatment group (Shapiro–Wilk test), and there is homogeneity of variances (Levene’s test), where the statistical significance is determined by a one-way analysis of variance (ANOVA) followed by Tukey’s post hoc comparison calculated with GraphPad Prism 5.0 software. Differences between groups were only deemed significant with a *p*-value of (*) 0.05, (**) 0.01, and (***) 0.001. All data are presented as the mean ± S.D.

## Results

### GBA1 K198E Mutation Decreases Lysosomal Glucocerebrosidase (GCase) Enzyme Activity, Increases GCase Expression Levels, and Increases Phosphorylation of LRRK2 (Ser935) and RAB10 (Thr73)

**Fig. 1:**
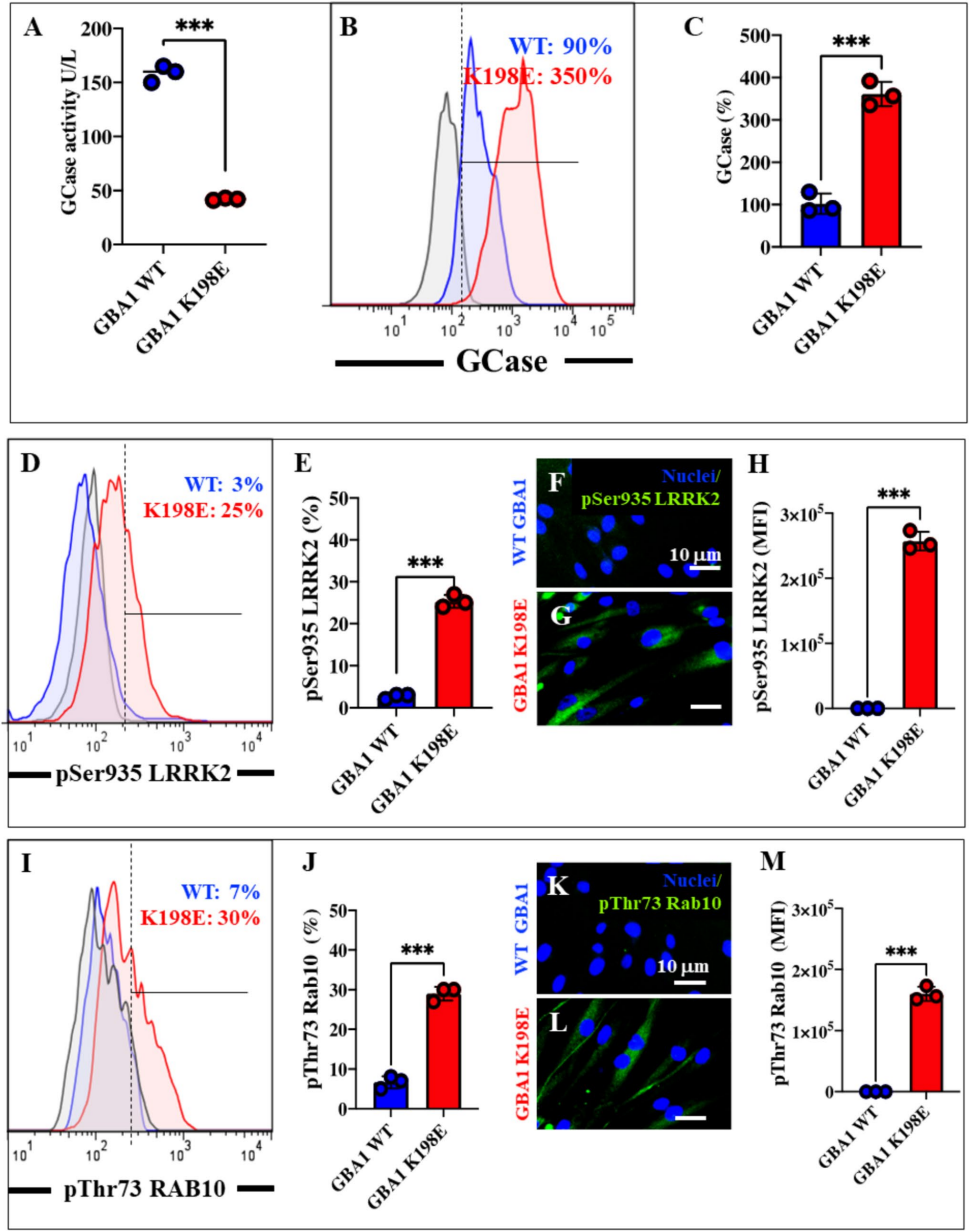
GBA1 K198E mutation alters glucocerebrosidase (GCase) substrate affinity, reduces enzymatic activity, and increases GCase levels and phosphorylation of LRRK2 (Ser935) and RAB10 (Thr73). **A** Enzyme activity of GCase in WT GBA1 (blue dots) and GBA1 K198E fibroblasts (red dots). **B** Protein expression levels of glucocerebrosidase (GCase) in WT GBA1 (blue curve) and GBA1 K198E fibroblasts (red curve) were analyzed via flow cytometry. **C** Quantification of GCase expression levels. Histogram numbers indicate the percentage of positive cellular populations for the tested marker. **D** Representative flow cytometry histogram analysis showing phosphorylated LRRK2 at Ser935 protein in WT (blue curve) and GBA1 K198E fibroblasts (red curve). **E** Quantitative analysis of p-Ser935 LRRK2 protein. **F-G** Immunofluorescence images showing p-Ser935 LRRK2 protein (green fluorescence) in WT GBA1 **(F)** and GBA1 K198E fibroblasts **(G)**. **H** Quantitative analysis of p-Ser935 LRRK2 protein in WT GBA1 (blue curve) and GBA1 K198E fibroblasts (red curve). GBA1 K198E fibroblasts show an endogenously high percentage of phosphorylated RAB10 at Thr73. **I** Representative flow cytometry histogram showing phosphorylated RAB10 at Thr73 in WT GBA1 (blue curve) and GBA1 K198E fibroblasts (red curve). **J** Quantitative analysis of p-Thr73 RAB10 levels**. K-L** Immunofluorescence images showing p-Thr73 RAB10 protein (green fluorescence) in WT GBA1 fibroblasts **(K)** and GBA1 K198E fibroblasts **(L). M** Quantitative analysis of phosphorylated RAB10 at Thr73 in WT GBA1 (blue) and GBA1 K198E fibroblasts (red). Numbers in histograms represent positive cellular population for the tested marker. The histograms and photomicrographs represent one out of three independent experiments (*n* = 3). The data are presented as mean ± SD of three independent experiments (dots in bar). One-way ANOVA followed by Tukey’s test. Statistically significant differences: ***p < 0.001. Image magnification 400 ×

Previous observations have shown that the GBA1 K198E variant significantly inactivates the catalytic enzymatic activity of GCase but does not affect protein expression levels (Perez-Abshana et al., 2024). Indeed, lysosomal GCase enzyme activity was reduced 3.76-fold in GBA1 K198E fibroblasts compared to WT GBA1 (Fig. 1A). As expected, mutant fibroblasts increased GCase protein expression levels 3.88-fold compared to control fibroblasts (Fig. 1B and C). We sought to determine the phosphorylation status of LRRK2 and RAB10 in WT GBA1 and K198E fibroblasts. As shown in Fig. 1D, mutant K198E fibroblasts increased p-Ser935 LRRK2 by + 733% compared to WT GBA1 (Fig. 1E). Similar results were obtained by fluorescence microscopy analysis (Fig. 1F–H). Similarly, mutant K198E fibroblasts increased p-Thr73 RAB by + 328% compared to WT GBA1 (Fig. 1I and J). Similar results were obtained by fluorescence microscopy analysis (Figs. 1K–M).

### PF-06447475 (PF-475) LRRK2 Kinase Inhibitor Reduced Levels of Phosphorylated (Ser935) LRRK2 in Fibroblasts Bearing GBA1 K198E Mutation

**Fig. 2:**
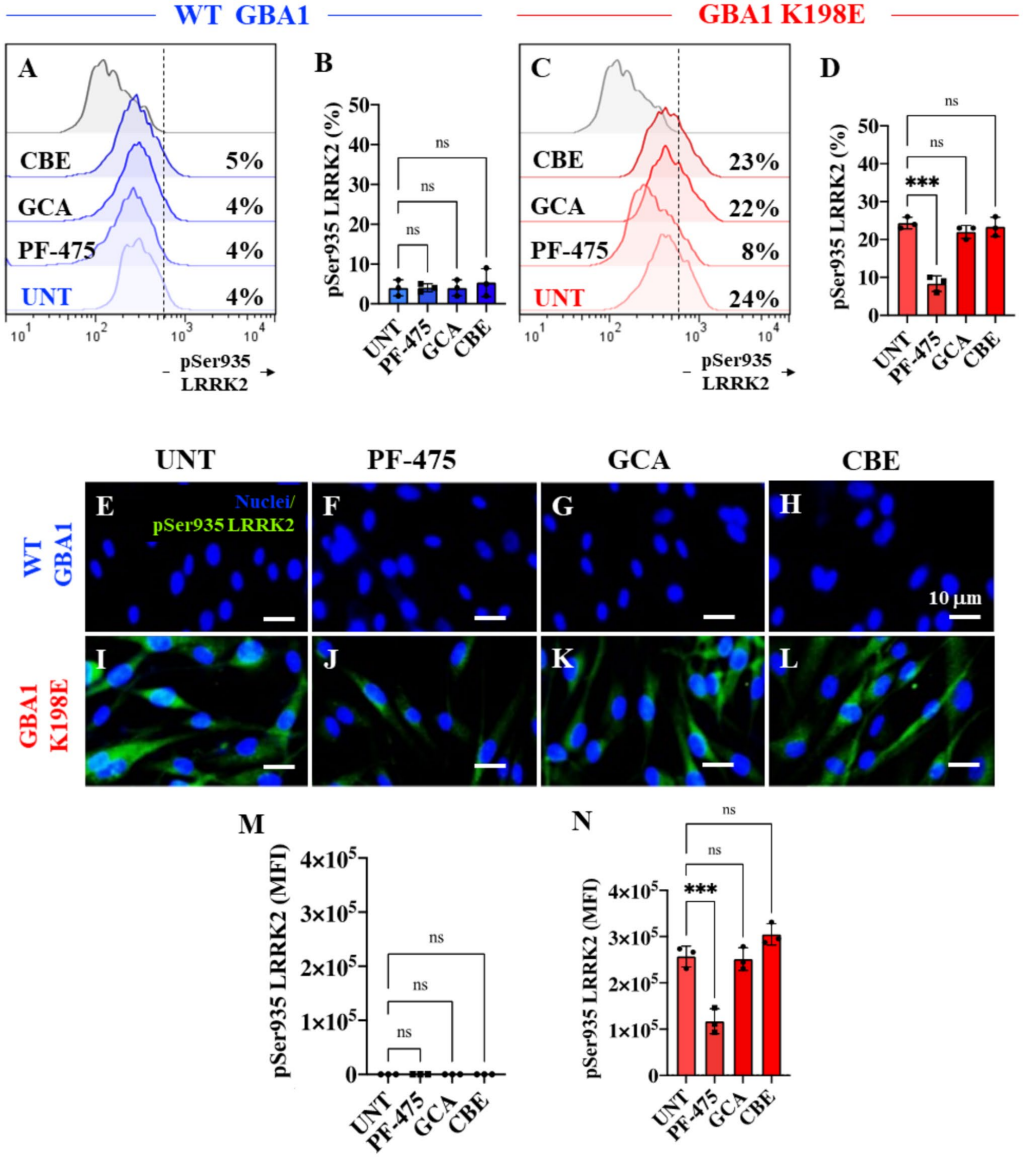
PF-06447475 (PF-475) LRRK2 kinase inhibitor reduced levels of phosphorylated (Ser935) LRRK2 in fibroblasts bearing GBA1 K198E mutation. **A** Representative flow cytometry histograms showing p-Ser935 LRRK2 in WT GBA1 fibroblasts untreated or treated with the LRRK2 inhibitor PF-475 (1 µM), the GCase stabilizer NCGC00188758 (GCA, 10 µM), or conduritol-B-epoxide (CBE, 10 µM) for 24 h. **B** Quantitative analysis of p-Ser935 LRRK2 levels in WT GBA1 fibroblasts. **C** Representative flow cytometry histograms showing p-Ser935 LRRK2 in GBA1 K198E fibroblasts untreated or treated with PF-475, GCA, or CBE for 24 h. **D** Quantitative analysis of p-Ser935 LRRK2 levels in GBA1 K198E fibroblasts. Immunofluorescence image of p-Ser935 LRRK2 (green fluorescence) and nuclei (blue fluorescence) in WT GBA1 **(E–H)** and GBA1 K198E **(I–L)** fibroblasts untreated **(E, I)** or treated with PF-475 **(F, J)**, GCA **(G, K),** or CBE **(H, L)** for 24 h. **M** Quantitative analysis of p-Ser935 LRRK2 in WT fibroblasts. **N** Quantitative analysis of p-Ser935 LRRK2 in GBA1 K198E fibroblasts. Numbers in histograms represent the percentage of the positive cell population for the tested marker. Results are from three independent experiments (*n* = 3) presented as mean ± SD. Statistical significance was assessed using one-way ANOVA followed by Tukey’s test; ***p < 0.001; ns = no significant. Image magnification 400 ×

Next, we evaluated the effect of the inhibitor PF-475, the GBA1 activator GCA, and the GBA1 inhibitor CBE in the context of p-LRRK2 status in WT GBA1 and K198E fibroblasts. Figure 2 shows that PF-475, GCA, and CBE did not affect the basal levels of p-Ser935 LRRK2 in WT GBA1 compared to untreated cells (Fig. 2A and B). In contrast, while PF-475 blocked p-Ser935 LRRK2 by − 67% in mutant fibroblasts compared to untreated mutant cells (Fig. 2C and D), neither GCA nor CBE affected the highly phosphorylated LRRK2 kinase status in K198E GBA1 fibroblasts (Fig. 2C and D). Similar results were obtained by fluorescence microscopy analysis (Fig. 2E–N).

### LRRK2 Kinase Inhibition Reduces Phosphorylated (Thr73) RAB10 in Fibroblasts Bearing GBA1 K198E Mutation

**Fig. 3:**
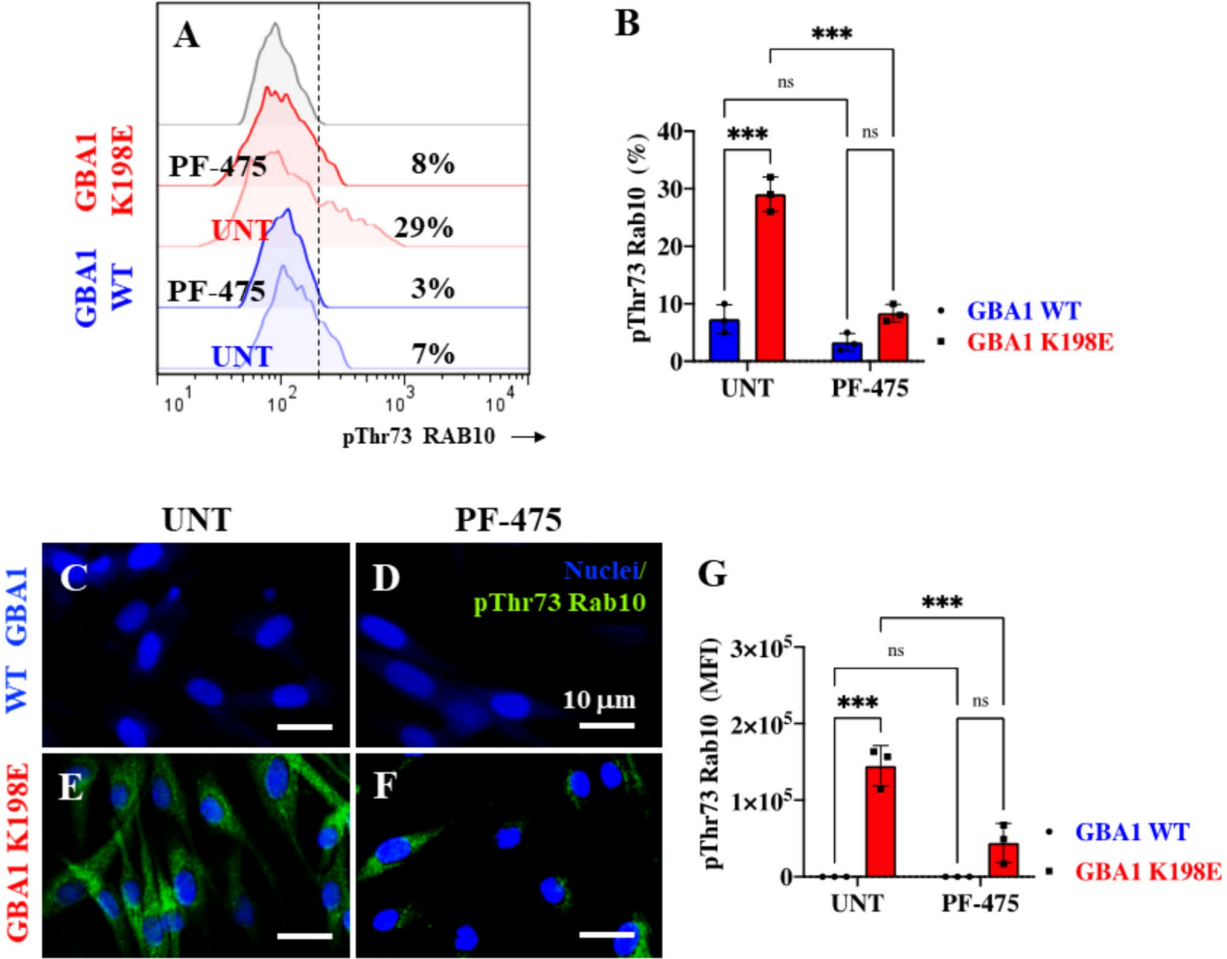
LRRK2 kinase inhibition reduced levels of phosphorylated (Thr73) RAB10 in fibroblasts bearing the GBA1 K198E mutation. **A** Representative flow cytometry histograms showing p-Thr73 RAB10 in WT and K198E GBA1 fibroblasts untreated or treated with LRRK2 inhibitor PF-475 (1 µM), **B** Quantitative analysis of p-Thr73 RAB10 levels in WT and K198E GBA1 fibroblasts. Immunofluorescence image of p-Thr73 RAB10 (green fluorescence) and nuclei (blue fluorescence) in WT GBA1 **(C-D)** and GBA1 K198E **(E-F)** fibroblasts untreated **(C, E)** or treated with PF-475 **(D, F)** for 24 h. **G** Quantitative analysis of p-Thr73 RAB10. Numbers in histograms represent the percentage of the positive cell population for the tested marker. Results are from three independent experiments (*n* = 3) presented as mean ± SD. Statistical significance was assessed using one-way ANOVA followed by Tukey’s test; ***p < 0.001; *ns* no significant. Image magnification 400 ×

The above observation prompted us to evaluate whether PF-475 also affected the phosphorylated status of RAB 10 in these fibroblasts. As shown in Fig. 3, PF-475 effectively reduced the p-Thr73 RAB 10 in WT GBA1 K198E and GBA1 fibroblasts by − 57% and − 72%, respectively (Fig. 3A and B). Similar results were obtained by fluorescence microscopy analysis (Figs. 3C–G).

### LRRK2 Kinase Inhibition Increases Glucocerebrosidase (GCase) Enzymatic Activity in Fibroblasts Bearing GBA1 K198E Mutation

**Fig. 4:**
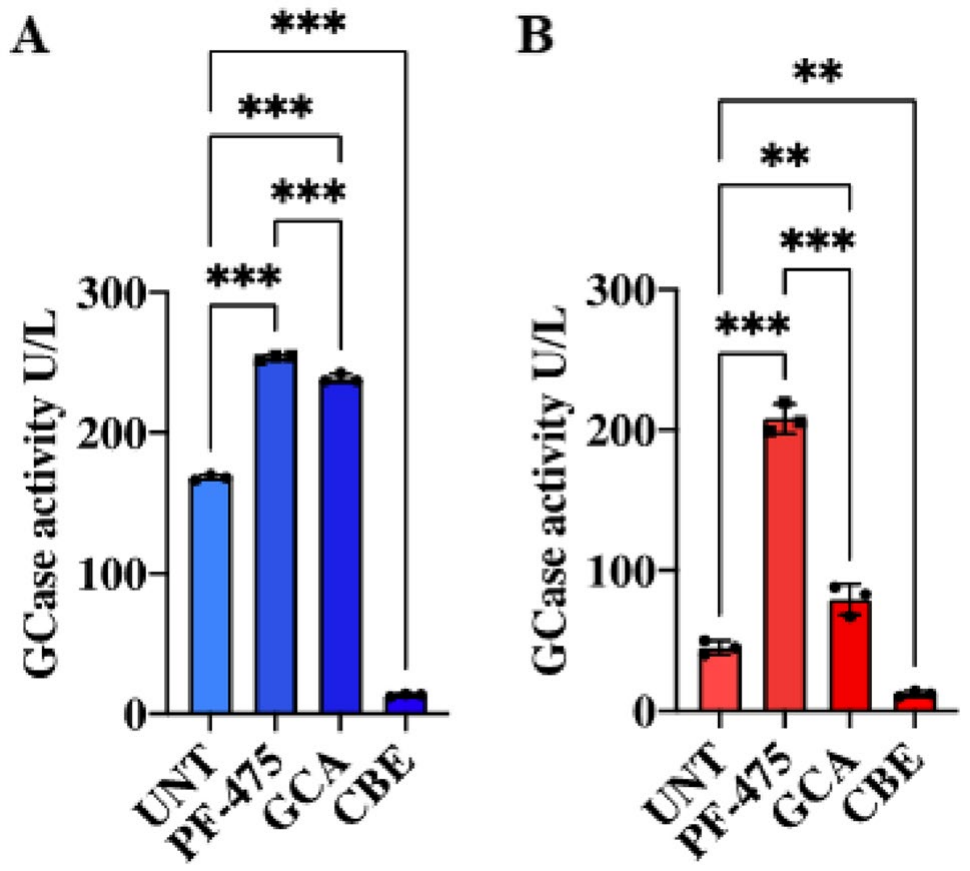
LRRK2 kinase inhibition increases glucocerebrosidase enzymatic activity in fibroblasts bearing GBA1 K198E mutation. **A** GCase enzyme activity in WT GBA1 fibroblasts (blue bars) treated for 24 h with the LRRK2 inhibitor PF-06447475 (1 µM), the GCase stabilizer NCGC00188758 (GCA, 10 µM, CID: 46,907,762) and conduritol-B-epoxide (CBE, 10 µM, CID: 136,345). **B** GCase enzyme activity in GBA1 K198E fibroblasts (red bar) under the same treatment conditions. Data are presented as mean ± SD of three independent experiments (*n* = 3). Statistical analysis was performed using one-way ANOVA followed by Tukey’s test; **p < 0.01 and ***p < 0.001

To further determine whether PF-475, GCA, or CBE affected the catalytic activity of GCase, WT and mutant cells were exposed to the inhibitors. Unexpectedly, PF-475 increased the GCase activity in both WT GBA1 (Fig. 4A) and K198E GBA1 cells (Fig. 4B) by + 51% and + 362%, respectively, whereas GCA and CBE were effective in enhancing or blocking CGase activity (Fig. 4A and B). The GCA was more effective in stimulating GCase activity in WT GBA1 (+ 76%) than in K198E GBA1 fibroblasts (+ 42%). Similarly, CBE reduced GCase activity more drastically in WT (− 92%) than in mutant fibroblasts (− 73%).

### LRRK2 Kinase Inhibition Prevents Lysosome Accumulation and Protects Mitochondrial Membrane Potential (∆Ψm) in Fibroblasts Bearing GBA1 K198E Mutation

**Fig. 5:**
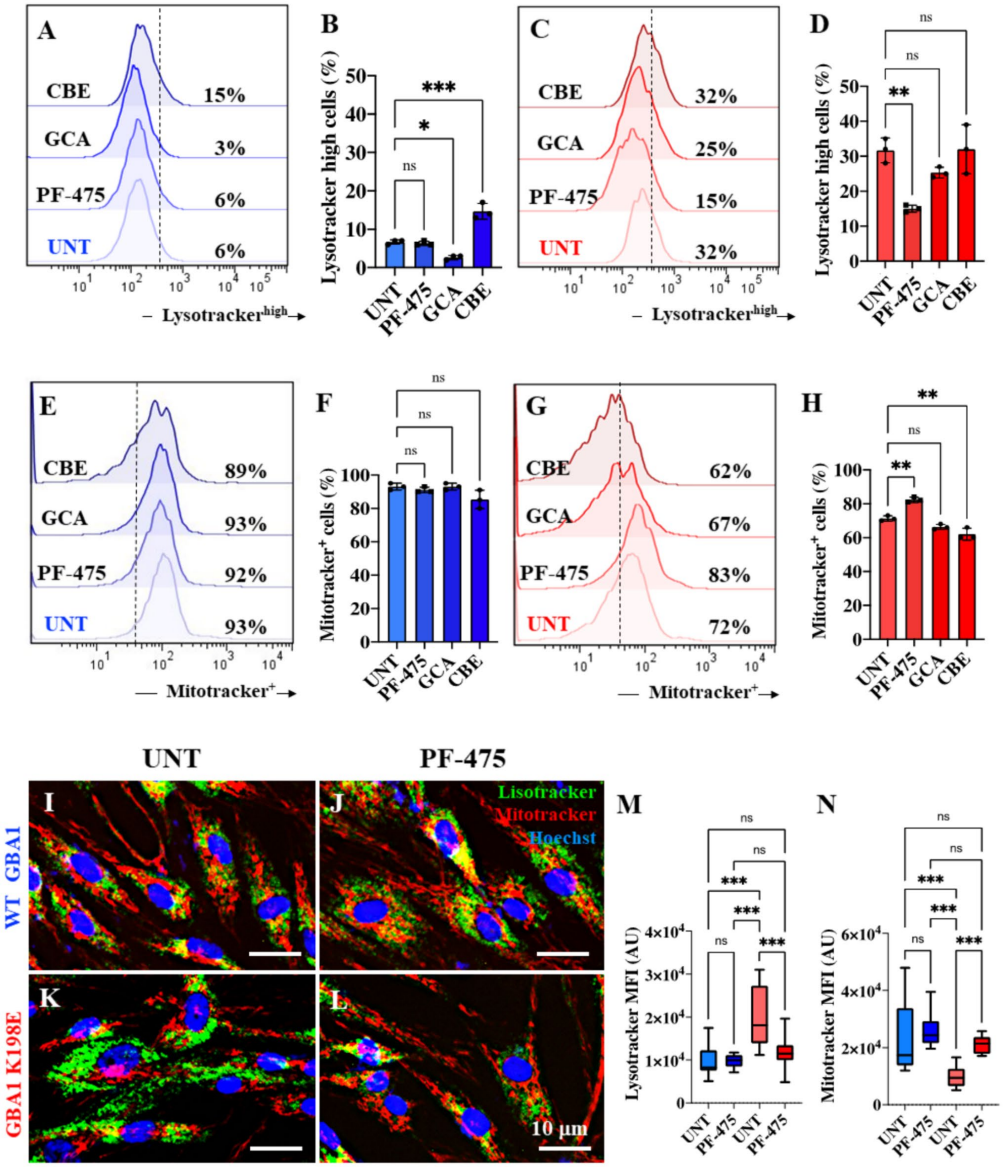
LRRK2 kinase inhibition prevents lysosome accumulation and protects mitochondrial membrane potential (∆Ψm) in fibroblasts bearing the GBA1 K198E mutation. **A** Representative flow cytometry histograms showing Lysotracker® staining in WT GBA1 fibroblasts (blue curve) untreated or treated with LRRK2 inhibitor PF-475 (1 µM), the GCase stabilizer NCGC00188758 (GCA, 10 µM), or conduritol-B-epoxide (CBE, 10 µM) for 24 h. **B** Quantitative analysis of Lysotracker® in WT GBA1 fibroblasts. **C** Representative flow cytometry histograms showing Lysotracker® staining in GBA1 K198E fibroblasts untreated or treated with PF-475, GCA, or CBE for 24 h. **D** Quantitative analysis of Mitotracker® in GBA1 K198E fibroblasts. **E** Representative flow cytometry histograms showing Mitotracker® staining in WT GBA1 fibroblasts (blue curve) untreated or treated with the LRRK2 inhibitor PF-475 (1 µM), the GCase stabilizer NCGC00188758 (GCA, 10 µM), or conduritol-B-epoxide (CBE, 10 µM) for 24 h. **F** Quantitative analysis of Mitotracker® in WT GBA1 fibroblasts. **G** Representative flow cytometry histograms showing Mitotracker® staining in GBA1 K198E fibroblasts untreated or treated with PF-475, GCA, or CBE for 24 h. **H** Quantitative analysis of Lysotracker® in GBA1 K198E fibroblasts. Representative fluorescence microscopy images showing Lysotracker® (green fluorescence), Mitotracker® (red fluorescence), and Hoechst (blue fluorescence) in WT GBA1 **(I-J)** and GBA1 K198E **(K-L)** fibroblasts untreated **(I, K)** or treated with PF-475 **(J, L)** for 24 h. **M** Quantification of the mean fluorescence intensity (MFI) for Lysotracker®. **N** Quantification of the mean fluorescence intensity (MFI) for Mitotracker®. Numbers in histograms represent the percentage of the positive cell population for the tested marker. Results are from three independent experiments (*n* = 3) presented as mean ± SD. Statistical significance was assessed using one-way ANOVA followed by Tukey’s test; *p < 0.05, **p < 0.01, ***p < 0.001; ns = no significant. Image magnification 400 ×

Previous observations have suggested that GBA1 K198E (lysosomal GCase) induces an increase in the acidification and accumulation of lysosomes and a moderate loss of ∆Ψm in mutant fibroblasts (Perez-Abshana et al., 2024). We further investigated whether PF-475, GCA, or CBE affected the lysosomes and ∆Ψm in WT and mutant GBA1 fibroblasts. Figure 5 shows that untreated WT showed a basal level of lysosomes (6%, Fig. 5A), whereas untreated GBA1 K198E showed an abnormally high level of lysosome accumulation (32%, Fig. 5C), i.e., compared to WT, the mutant fibroblast showed an endogenous increase of lysosome accumulation by + 433%. When cells were treated with PF-475, the inhibitor did not affect the basal level of lysosomes in WT GBA1 fibroblasts but significantly reduced the accumulation of lysosomes in GBA1 K198E fibroblasts by − 53%. The activator GCA was effective in reducing the basal level and accumulation of lysosomes in WT (Fig. 5A and B) and mutant fibroblasts (Fig. 5C and D) by − 50% and − 22%, respectively, whereas the inhibitor GCase CBE induced a marked increase in the accumulation of lysosomes in WT by + 150% (Fig. 5A and B) but was ineffective in mutant fibroblasts (Fig. 5C and D). Interestingly, none of the agents altered the ∆Ψm in WT (Fig. 5E and F), but PF-475 increased ∆Ψm in GBA1 K198E by + 15% (Fig. 5G and H). While GCA-treated mutant fibroblasts showed no statistical differences in ∆Ψm and untreated GBA1 K198E, CBE slightly decreased ∆Ψm by − 14% in mutant fibroblasts (Fig. 5G and H). Fluorescence microscopy analysis confirmed that PF-475 was harmless in WT GBA1 fibroblasts (Fig. 5I, J, M and N), but effectively decreased the accumulation of lysosomes and increased ∆Ψm in GBA1 K198E (Fig. 5K–N).

### LRRK2 Kinase Inhibition Reduced Levels of Phosphorylated (Ser129) α-Synuclein (α-Syn) in Fibroblasts Bearing GBA1 K198E Mutation

**Fig. 6:**
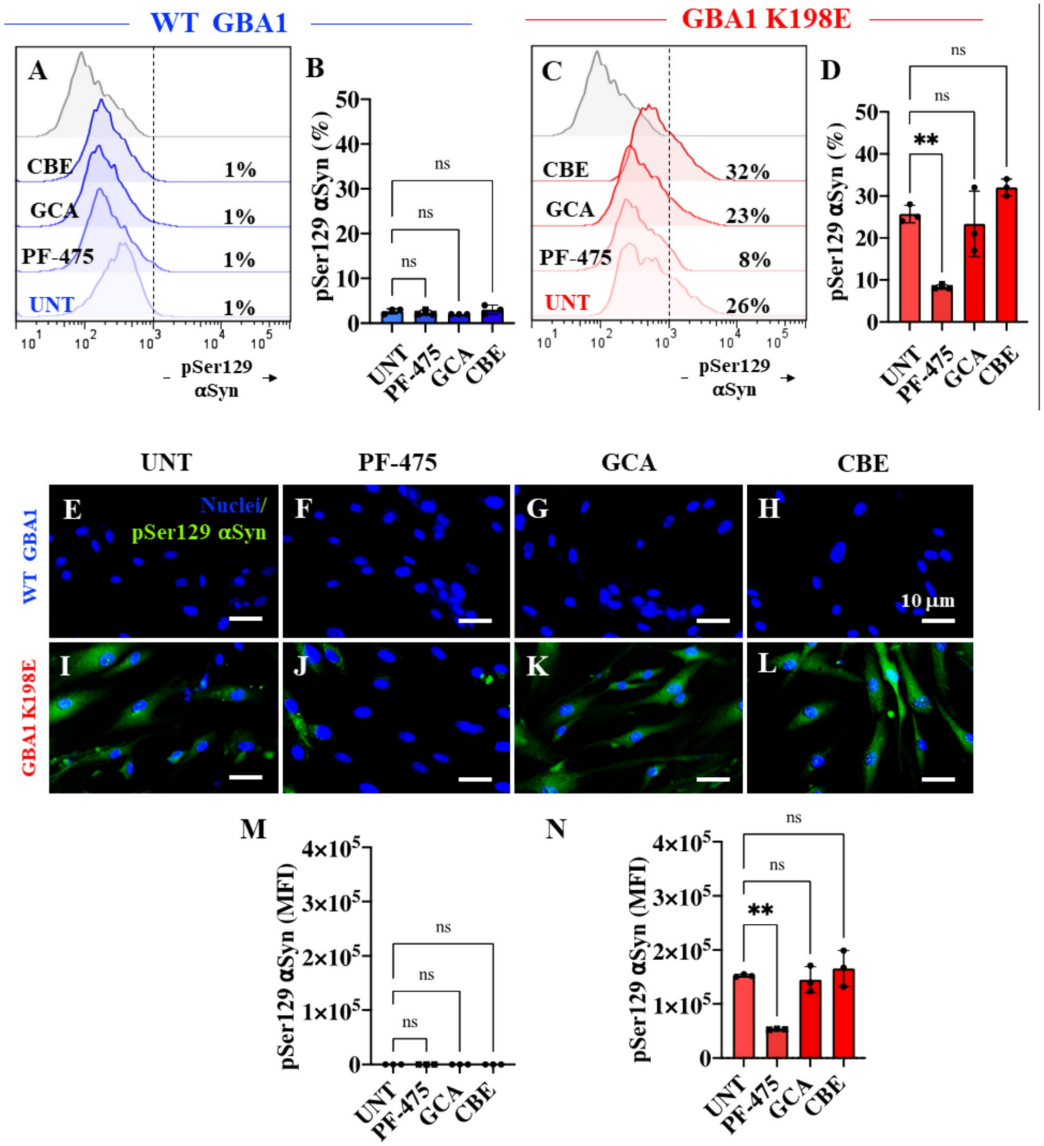
LRRK2 kinase inhibition reduced levels of phosphorylated (Ser129) α-synuclein (α-Syn) in fibroblasts bearing the GBA1 K198E mutation. **A** Representative flow cytometry histograms showing p-Ser129 α-Syn in WT GBA1 fibroblasts untreated or treated with the LRRK2 inhibitor PF-475 (1 µM), the GCase stabilizer NCGC00188758 (GCA, 10 µM), or conduritol-B-epoxide (CBE, 10 µM) for 24 h. **B** Quantitative analysis of p-Ser129 α-Syn levels in WT GBA1 fibroblasts. **C** Representative flow cytometry histograms showing p-Ser129 α-Syn in GBA1 K198E fibroblasts untreated or treated with PF-475, GCA, or CBE for 24 h. **D** Quantitative analysis of p-Ser129 α-synuclein levels in GBA1 K198E fibroblasts. Immunofluorescence image of p-Ser129 α-Syn (green fluorescence) and nuclei (blue fluorescence) in WT GBA1 **(E–H)** and GBA1 K198E **(I-L)** fibroblasts untreated **(E, I)** or treated with PF-475 **(F, J)**, GCA **(G, K),** or CBE **(H, L)** for 24 h. **M** Quantitative analysis of p-Ser129 α-Syn in WT fibroblasts. **N** Quantitative analysis of p-Ser129 α-Syn in GBA1 K198E fibroblasts. Numbers in histograms represent the percentage of the positive cell population for the tested marker. Results are from three independent experiments (*n* = 3) presented as mean ± SD. Statistical significance was assessed using one-way ANOVA followed by Tukey’s test; **p < 0.01, ns = no significant). Image magnification 400 ×

We investigated whether PF-475, GCA, or CBE altered the p-Ser129 α-Syn in fibroblasts. As shown in Fig. 6, neither PF-475, GCA, nor CBE affected the basal level of p-Ser129 α-Syn (1%) in WT fibroblasts (Fig. 6A-B). PF-475 induced a − 69% decrease in p-Ser129 α-Syn by in GBA1 K198E fibroblasts (Fig. 6C and D); in contrast, no significant differences in p-Ser129 α-Syn were found when GBA1 K198E fibroblasts treated with GCA and CBE were compared with untreated mutant cells (Fig. 6C and D). Similar results were obtained by fluorescence microscopy analysis (Fig. 6E–N).

### LRRK2 Kinase Inhibition Reduced Levels of Oxidized DJ-1 and Cleaved Caspase-3 in Fibroblasts Bearing GBA1 K198E Mutation

**Fig. 7:**
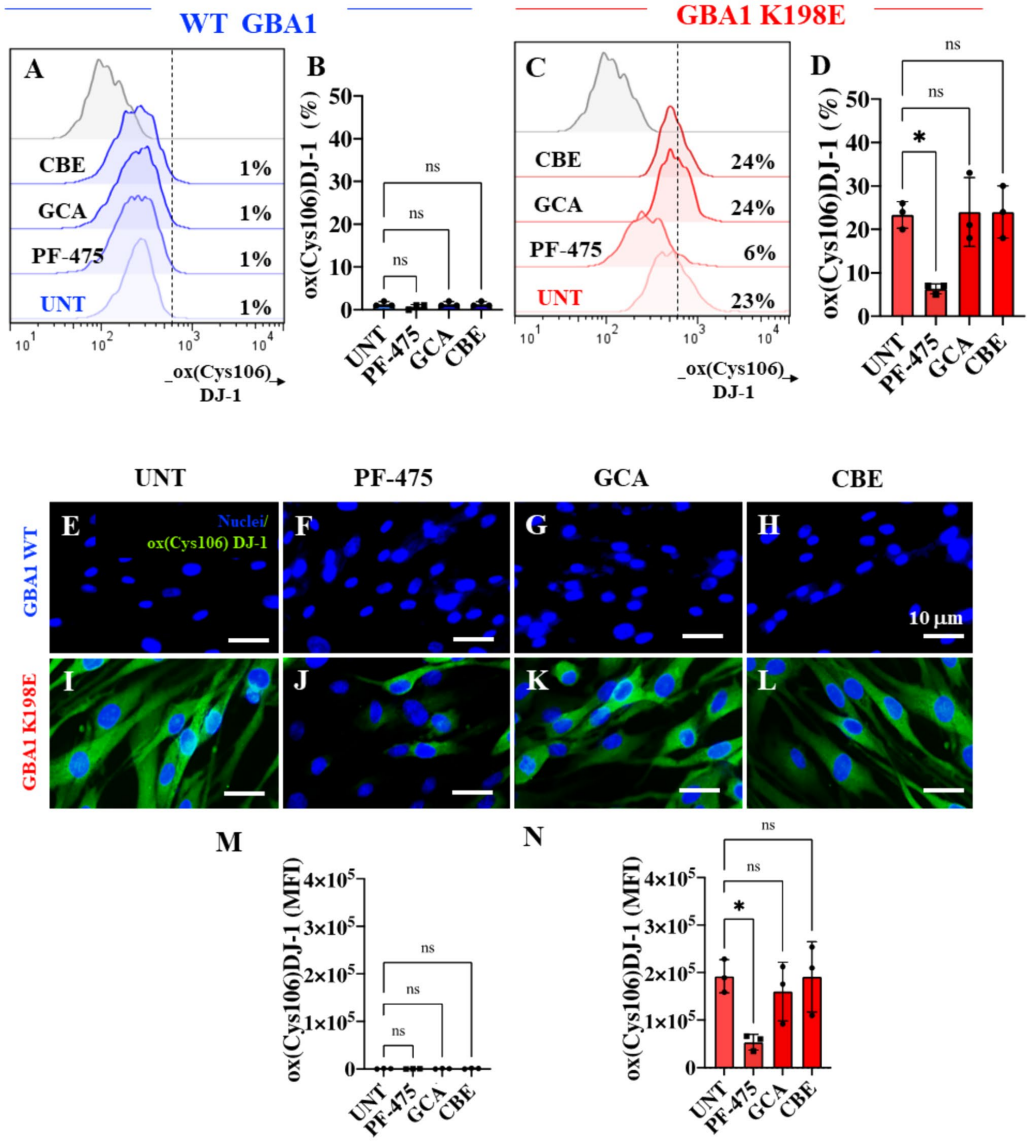
LRRK2 kinase inhibition reduced levels of oxidized (Cys106) DJ-1 (ox(Cys106)DJ-1) in fibroblasts bearing GBA1 K198E mutation. **A** Representative flow cytometry histograms showing ox(Cys106)DJ-1 in WT GBA1 fibroblasts untreated or treated with the LRRK2 inhibitor PF-475 (1 µM), the GCase stabilizer NCGC00188758 (GCA, 10 µM), or conduritol-B-epoxide (CBE, 10 µM) for 24 h. **B** Quantitative analysis of ox(Cys106)DJ-1 levels in WT GBA1 fibroblasts. **C** Representative flow cytometry histograms showing ox(Cys106)DJ-1 in GBA1 K198E fibroblasts untreated or treated with PF-475, GCA, or CBE for 24 h. **D** Quantitative analysis of ox(Cys106)DJ-1 levels in GBA1 K198E fibroblasts. Immunofluorescence image of ox(Cys106)DJ-1 (green fluorescence) and nuclei (blue fluorescence) in WT GBA1 **(E–H)** and GBA1 K198E **(I-L)** fibroblasts untreated **(E, I)** or treated with PF-475 **(F, J)**, GCA **(G, K),** or CBE **(H, L)** for 24 h. **M** Quantitative analysis of ox(Cys106)DJ-1 in WT fibroblasts. **N** Quantitative analysis of ox(Cys106)DJ-1 in GBA1 K198E fibroblasts. Numbers in histograms represent the percentage of the positive cell population for the tested marker. Results are from three independent experiments (*n* = 3) presented as mean ± SD. Statistical significance was assessed using one-way ANOVA followed by Tukey’s test; *p < 0.05, *ns* no significant. Image magnification 400 ×

**Fig. 8:**
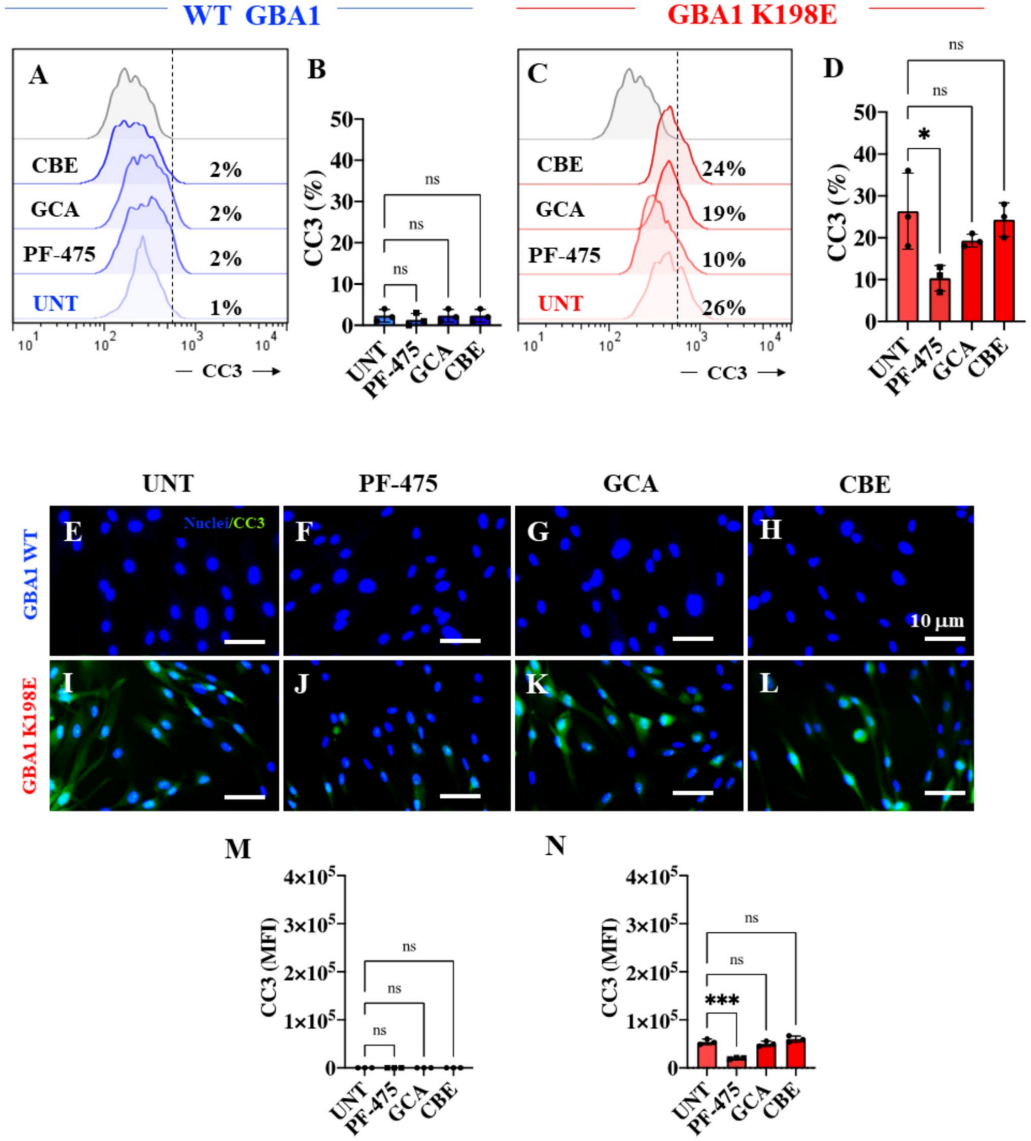
LRRK2 kinase inhibition reduced levels of cleaved (active) caspase-3 (CC3) in fibroblasts bearing the GBA1 K198E mutation. **A** Representative flow cytometry histograms showing CC3 in WT GBA1 fibroblasts untreated or treated with the LRRK2 inhibitor PF-475 (1 µM), the GCase stabilizer NCGC00188758 (GCA, 10 µM), or conduritol-B-epoxide (CBE, 10 µM) for 24 h. **B** Quantitative analysis of CC3 levels in WT GBA1 fibroblasts. **C** Representative flow cytometry histograms showing CC3 in GBA1 K198E fibroblasts untreated or treated with PF-475, GCA, or CBE for 24 h. **D** Quantitative analysis of CC3 levels in GBA1 K198E fibroblasts. Immunofluorescence image of CC3 (green fluorescence) and nuclei (blue fluorescence) in WT GBA1 **(E–H)** and GBA1 K198E **(I-L)** fibroblasts untreated **(E, I)** or treated with PF-475 **(F, J)**, GCA **(G, K),** or CBE **(H, L)** for 24 h. **M** Quantitative analysis of CC3 in WT fibroblasts. **N** Quantitative analysis of CC3 in GBA1 K198E fibroblasts. Numbers in histograms represent the percentage of the positive cell population for the tested marker. Results are from three independent experiments (*n* = 3) presented as mean ± SD. Statistical significance was assessed using one-way ANOVA followed by Tukey’s test; *p < 0.05, ***p < 0.001; *ns* no significant. Image magnification 400 ×

Finally, we sought to determine the levels of DJ-1 protein oxidation as a sign of OS and CC3 as indicative of apoptosis. Figure 7 shows that while the detection of DJ-1 Cys106-SO_3_ (Fig. 7A and B) and CC3 (Fig. 8A and B) in untreated WT GBA1 was low (~ 1%), K198E fibroblasts showed a significant increase in DJ-1 Cys106-SO_3_ (Fig. 7C and D, + 2200%) and CC3 (Fig. 8C and D, + 2500%). Strikingly, when fibroblasts were treated with PF-475, WT GBA1 cells were unaffected (Figs. 7A-D), but K198E fibroblasts showed a significant reduction in DJ-1 Cys106-SO_3_ (Fig. 7C and D, − 74%) and CC3 (Fig. 8C and D, − 61%). GCA and CBE did not affect oxidized DJ-1 and CC3 in either WT or GBA1 K198E fibroblasts. Similar results were obtained by fluorescence microscopy analysis (Figs. 7E–N and 8E–N).

## Discussion

**Fig. 9:**
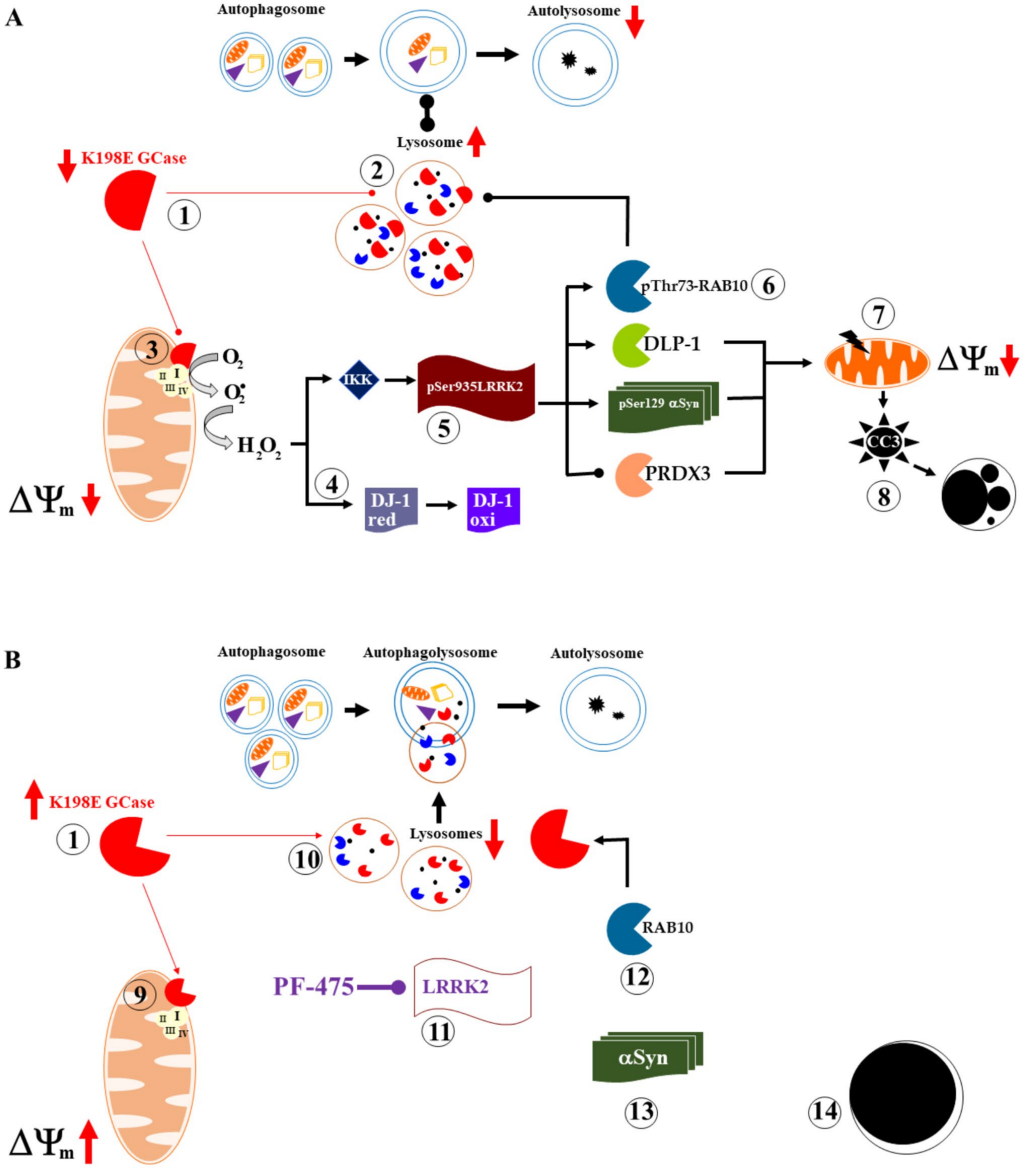
Schematic representation of the cellular effects of GBA1 K198E variant in skin fibroblasts: A pathogenic phenotype reverted by the inhibitor LRRK2 PF-06447475 (PF-475). For explanation, see text

Here, we have confirmed that GBA1 K198E fibroblasts, but not WT fibroblasts, displayed a significant reduction in the catalytic activity of heterozygous mutant GCase without affecting its expression levels; increased phosphorylated LRRK2 at Ser935 along with phosphorylated α-Syn at pathological residue Ser129; decreased the mitochondrial membrane potential (ΔΨm); increased the oxidized DJ-1 in the form of DJ-1 Cys106-SO_3_, as evidence of OS; increased the accumulation of lysosomes; and increased the executioner apoptotic protein caspase-3 (CC3) (Perez-Abshana et al., 2024). In addition, we found that mutant fibroblasts exhibited increased phosphorylation of RAB 10 protein at residue Thr73 compared to WT. Based on these observations, a two-step concurrent mechanistic scenario by which the heterozygous GBA1 K198E mutation causes deterioration in skin fibroblasts (or in neuronal cells) is illustrated in Fig. 9A. First, in at least one GBA1 allele, the enzymatic modification of GCase by K198E (step 1) results in nearly undigested GlcCer. This results in the accumulation of lysosomes (step 2), which affects the autophagosome and autolysosome production line. Autophagosomes and lysosomes accumulate abnormally when K198E GCase is present. Thus, in skin fibroblasts, K198E GCase induces a severely impaired autophagy-lysosomal pathway (ALP). Second, dysfunction of mitochondrial Complex I due to improper interactions with K198E GCase (step 3) (Baden et al., 2023) allows electrons to leak out, which are taken up by molecular oxygen (O₂). The reduction of oxygen results in the formation of anionic superoxide radicals (O₂⁻), which are then dismutated to H_2_O_2_. This non-radical agent, which serves as a second messenger (Marinho et al., 2014), in turn oxidizes the OS sensor protein DJ-1 Cys106-SOH to DJ-1 Cys106-SO_3_ (-sulfonic group, step 4) (Kinumi et al., 2004) and could activate LRRK2 kinase by directly increasing its autophosphorylation, e.g., at Tyr1967 (Kamikawaji et al., 2009), Ser2032, and Tyr2035 (Li et al., 2010; West et al., 2007), or indirectly via phosphorylation of Ser910 and Ser935 (step 5) by the inhibitor of nuclear factor-κB (IκB) kinase (IKK) complex (Dzamko et al., 2012). Once active, LRRK2 kinase phosphorylates four major targets. The fission mitochondrial dynamin-like protein (DLP-1) (Wang et al., 2012), α-Syn at residue Ser129 (Qing et al., 2009), PRDX3 (Angeles et al., 2011), and RAB10 at residue Thr73 (step 6) (Kuwahara et al., 2020). These proteins may induce or contribute to mitochondrial depolarization (step 7, e.g., low ΔΨm), aberrant accumulation of α-Syn, increased production of H₂O₂, and p-Thr73 RAB10-induced lysosomal GCase dysfunction. All of these actions can induce or contribute to the activation of caspase-3 (CASP3) to cleaved caspase-3 (CC3, step 8), which induces nuclear fragmentation. Each of these indicators is a common indication of the intrinsic pathway of apoptosis (Lossi, 2022).

We report for the first time that the LRRK2 inhibitor PF-475 (Fig. 9B) not only restores GCase enzyme activity (step 1) but also restores vital cellular functions such as increasing the mitochondrial membrane potential (ΔΨm, step 9), significantly decreasing DJ-1 Cys106-SO_3_, reducing lysosome accumulation (step 10), and reducing CC3 in GBA1 K198E fibroblasts. Furthermore, in addition to a significant reduction in p-Ser935 LRRK2 kinase (step 11), PF-475 reduced p-RAB 10 (step 12) and p-Ser129 α-Syn (step 13), thereby increasing cell survival (step 14). Taken together, these observations suggest that LRRK2 is a critical signaling kinase in the pathogenic mechanism associated with the lysosomal GBA1/GCase K198E variant.

In agreement with Ysselstein and co-workers (2019), we found that inhibition of LRRK2 kinase activity results in increased GCase activity in fibroblasts with GBA1 mutations. Furthermore, the increase in GCase is sufficient to reduce the accumulation of lysosomes, oxidized DJ-1, and p-α-Syn in GBA1 K198E fibroblasts. We also found that p-Thr73 RAB10 was phosphorylated simultaneously with p-LRRK2. This observation suggests that RAB10 may be a mediator of LRRK2 regulation of GCase activity (Ysselstein et al., 2019). These results suggest an important role for LRRK2 as a regulator of lysosomal GCase activity. But how does inhibition of LRRK2 restore K198E GCase to normal enzymatic activity? This is still an open question. One possible explanation is that, under normal conditions, GCase activation requires the facilitator protein saposin C (SAPC) (Sun et al., 2003) and RAB10 (Ysselstein et al., 2019). Indeed, using multiscale molecular dynamics simulations and deep learning, Romero and coworkers (2019) have proposed that conformational changes in loops 1–5 (residues 311–319, 345–349, 394–399, 237–248, and 283–288) at the entrance of the substrate binding site of GCase (active site, catalytic residue E340 (catalytic nucleophile), and E235 (acid–base residue)), stabilized by direct interactions with SAPC, regulate substrate accessibility. The SAPC binding site on GCase is proposed to be located between domain III loops 1 (H311-P319) and 2 (S345-S351) at the active site entrance, helix 6 (K321-L330), helix 7 (W357-L372), and domain II (T43-S45, Q440-D445, L461-S465, and Y487) (Romero et al., 2019). Therefore, loss of such interactions induced by K198E and another common mutation (N370S, L444P) or p-Thr73 RAB10 results in destabilization of the complex and reduced GCase activation. Indeed, the K198E variant significantly reduces the catalytic enzymatic activity of GCase (~ − 70%) by replacing a positively charged (basic) lysine (K) with a negatively charged glutamic acid (E), which alters the three-dimensional structure of the enzyme (Sotomayor-Vivas et al., 2022). In addition, in silico molecular docking analysis suggested that the K198E variant almost completely disabled the catalytic ligand-substrate binding pocket (e.g., GlcSph) (Perez-Abshana et al., 2024). However, the variant increased protein expression levels, most likely as a result of an enzymatic compensatory functional mechanism. We find that the enzyme deficiency of K198E GCase is rescued by either the activator GCA (NCGC00188758) or PF-475. One possibility is that GCA or dephosphorylated RAB10 stabilizes the GCase-SAP complex, increasing substrate fit at the entrance of the substrate-binding site and thereby increasing GCase activity. However, whether dephosphorylated RAB10 interacts directly or indirectly with the protein–protein complex or facilitates GCase-substrate interactions in the intra-lysosomal membrane environment requires further investigation.

We report for the first time that the enzymatic enhancer GCA (a non-inhibitory chaperone) (Patnaik et al., 2012) restores GCase enzyme activity and reduces lysosome accumulation in GBA1 K198E fibroblasts (Mazzulli et al., 2016); however, GCA was unable to reduce either α-Syn or the OS-associated molecular events (e.g., p-LRRK2, oxDJ-1, ∆Ψm, CC3) (Mazzulli et al., 2016). Interestingly, the GCase inhibitor conduritol-β-epoxide (CBE), used as an internal control, significantly reduced GCase and kept the other pathological markers largely unchanged in GBA1 K198E, but reduced GCase activity and increased lysosome accumulation only in WT GBA1 fibroblasts. Taken together, these observations reinforce the notion that pharmacological inhibitors or activators of GCase only affect ALP, whereas genetic mutations in GCase (e.g., K198E) induce alteration of ALP and apoptosis signaling via GBA1 K198E-lysosome and GBA1 K198E-mitochondria > p-LRRK2. These observations may explain why the use of small chemical chaperones, either with inhibitory, non-inhibitory or mixed types, has not been fully effective for the treatment of PD (Menozzi et al., 2023).

Similar to the other LRRK2 inhibitor, DNL201 (Jennings et al., 2022), PF-475 inhibited LRRK2 kinase activity as evidenced by decreased phosphorylation of both LRRK2 at p-Ser935 and p-Thr73 RAB10, a direct substrate of LRRK2. Despite the fact that PF-475 is one of the most brain-penetrant antagonists, showing good tolerability at a relatively high dose (65 mg/kg) in a 2 week in vivo toxicology study (Henderson et al., 2015), there are no data yet to determine whether this inhibitor could be effective in PD (Jennings et al., 2022). Clinical trials are now underway for one antisense oligonucleotide (BIIB-094) and four LRRK2 kinase inhibitors (DNL201, WXWH0226, NEU-723, and BIIB122) (Hu et al., 2023). Moreover, type I and II kinase inhibitors await further investigation in clinical trials (Zhu et al., 2024). Our findings therefore suggest that LRRK2 inhibition is a potential disease-modifying therapeutic strategy for the PD-GBA1 K198E variant.

In conclusion, we have shown that the specific inhibitor LRRK2 PF-475 restores GCase activity through downregulation of RAB10, reduces lysosome accumulation, and reverses the GBA1 K198E-induced PD phenotype (e.g., p-Ser129 α-Syn, DJ-Cys106-SO_3_, low ∆Ψm, and CC3-positive cells) in skin fibroblasts. Although several GBA1 inhibitors have entered clinical trials for PD with or without GBA1 variants (Menozzi et al., 2023), our findings suggest that LRRK2 inhibitors already in clinical trials (Hu et al., 2023) could be tested in PD patients with (e.g., K198E) or without GBA1 variants.

## Data Availability

No datasets were generated or analyzed during the current study.
